# Highly efficient redox reaction between potassium permanganate and 3,3′,5,5′-tetramethylbenzidine for application in hydrogen peroxide based colorimetric assays†

**DOI:** 10.1039/c8ra07758d

**Published:** 2019-01-14

**Authors:** Ying Sun, Hui Liu, Xionghong Tan, Zheng Li, Yanlin Du, Aixian Zheng, Xiaolong Liu, Niancai Peng

**Affiliations:** College of Life Science, Fujian Agriculture and Forestry University Fuzhou 350002 P. R. China; Fujian Institute of Research on the Structure of Matter, Chinese Academy of Sciences Fuzhou 350002 P. R. China zax040500273@126.com xiaoloong.liu@gmail.com; The United Innovation of Mengchao Hepatobiliary Technology Key Laboratory of Fujian Province, Mengchao Hepatobiliary Hospital of Fujian Medical University Fuzhou 350025 P. R. China; State Key Laboratory for Manufacturing Systems Engineering, School of Mechanical Engineering, Xi'an Jiaotong University Xi'an 710054 P. R. China pnc@mail.xjtu.edu.cn; Fifth People's Hospital Ganzhou City Jiangxi Province China

## Abstract

Potassium permanganate (KMnO_4_) is one of the most important oxidants, which plays important roles in many fields. Here, we found that KMnO_4_ could directly induce the oxidation of 3,3′,5,5′-tetramethylbenzidine (TMB) to generate an oxidized product with a color change. This redox reaction is highly efficient, and 1 μM KMnO_4_ is enough to cause detectable changes in the absorbance signal. Meanwhile, this reaction is very fast and the generated blue product can stabilize for a relatively long period, which has great advantages in practical applications. Since hydrogen peroxide (H_2_O_2_) is able to react with KMnO_4_ under acidic conditions, the KMnO_4_-TMB system can be used for the detection of H_2_O_2_; the absorbance signal induced by 5 μM H_2_O_2_ can be easily detected in this method. Meanwhile, the KMnO_4_-TMB system can also be used for the detection of glucose by monitoring the generation of H_2_O_2_, which is the main product of glucose oxidation; this method permits detection of concentrations as low as 10 μM glucose, and the sensitivity is comparable to or higher than most peroxidase mimetic based methods, but avoiding the preparation and storage of the nanomaterials. Furthermore, the KMnO_4_-TMB system can even be used for analyzing glucose in serum samples, which can also be expected to be used in immunoassays.

## Abstract 2

The redox reaction between potassium permanganate and 3,3′,5,5′-tetramethylbenzidine is fast and highly efficient, which can be used for different biosensing.

## Graphical abstract

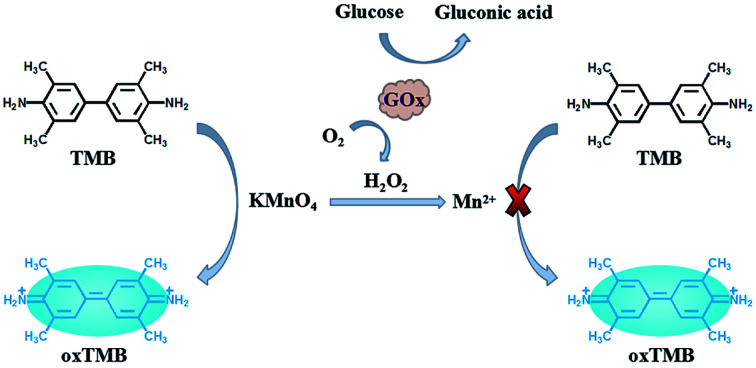

## Introduction

The development of simple, fast and cost-effective bioassays for the detection of disease related biomarkers plays important roles in biomedical application. Natural enzymes show high catalytic activity and substrate specificity, which have been applied to various fields, such as food safety, environmental analysis, biotechnology and biomedicine. For example, horseradish peroxidase (HRP) has been widely used in enzyme linked immunosorbent assay (ELISA), which can be conjugated with antibodies for the detection of specific proteins through an HRP mediated color reaction. However, the catalytic activity of natural enzymes is highly related to the environmental conditions during measurement, such as pH and temperature. Furthermore, the strict conditions and high cost of their preparation and storage could further limit their applications.^1–3^

To overcome these shortcomings, recent efforts have been devoted to develop simple and efficient enzymatic mimics. Up to now, a variety of nanomaterials have been reported to exhibit peroxidase-like catalytic activities, such as magnetic nanomaterials,^4,5^ carbon nanomaterials,^6–8^ metal–organic frameworks (MOFs),^9,10^ and so on.^1,11–16^ Compared with natural enzymes, these peroxidase mimetics exhibit obviously improved stability and design flexibility, which can be applied in different biosensing applications. It is also reported that some nanomaterials^17^ such as V_2_O_5_ nanosheets,^18^ MnO_2_ nanosheets^19,20^ and cobalt oxyhydroxide nanoflakes,^21,22^ exhibited intrinsic oxidation ability, which can directly induce the oxidation of 3,3′,5,5′-tetramethylbenzidine (TMB) to generate the oxidant product with color change. These reactions are fast and highly efficient, which can be further used for the detection of many biomolecules.

Although lot of efforts have been devoted to the nanomaterial based methods, there are still some deficiencies that limit their applications, such as complicated preparation, the uniformity and stability of the nanomaterials. Meanwhile, the catalytic sites of the enzymatic mimics are always on their surfaces, which might be easily blocked by nonspecific proteins or other biomolecules. In this way, the catalytic activities of the enzymatic mimics would be significantly reduced, and thus further reduce the sensitivity of these enzymatic mimic based sensors.

Potassium permanganate (KMnO_4_) is one of the most important oxidants, which plays important roles in many fields. In this paper, we found that KMnO_4_ can directly induce the oxidation of TMB to generate the oxidized product with significant color change. Meanwhile, the reaction is very fast and the generated blue product can be stable for a relatively long period. It is well known that hydrogen peroxide (H_2_O_2_) is able to reaction with KMnO_4_ in acidic conditions, and thus causing the consumption of KMnO_4_. In this way, the KMnO_4_-TMB system can be used for the detection of H_2_O_2_. Meanwhile, the KMnO_4_-TMB system can be also used for the indirectly detection of other biomolecules by monitoring the generation of H_2_O_2_.

**Scheme 1:**
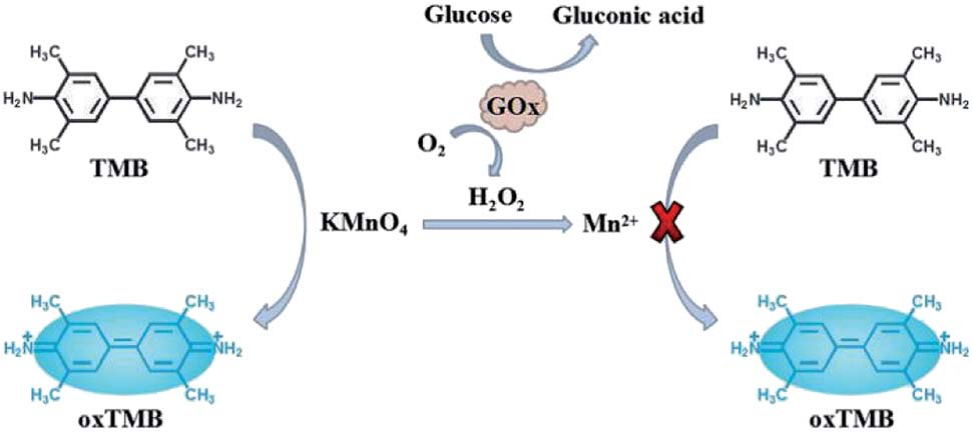
Highly efficient redox reaction between KMnO_4_ and TMB for application in H_2_O_2_ based colorimetric detection of glucose.

Glucose is the main energy source for human body, which plays important roles in cell growth. The concentration of glucose in blood is closely associated with hypoglycemia or diabetes, which can be also used for monitoring many other diseases. Thus, accurate measurement of glucose is of great importance.^23–25^ As we know, glucose oxidase (GOx) can catalyze the oxidation of glucose in the presence of oxygen, accompanying the formation of gluconic acid and H_2_O_2_.^6,8^ Thus, the proposed KMnO_4_-TMB system also can be used for the detection of glucose by monitoring the generation of H_2_O_2_. Scheme 1 depicts the principle of KMnO_4_-TMB system for glucose detection.

## Experimental

### Materials and apparatus

TMB, β-lactose, ascorbic acid and uric acid were purchased from Aladdin Reagent Co. Ltd. (China). Glucose, sucrose and galactose were purchased from Sigma-Aldrich Chemical Co. (USA). GOx was purchased from J & K Chemical Ltd. (China). All other reagents were of analytical grade and obtained from Sinopharm Chemical Reagent co. Ltd. (China), such as KMnO_4_, α-lactose and H_2_O_2_. Ultrapure water used in this experiment was obtained from a Millipore water purification system (Milli-Q). The absorption signals in this experiment were measured by microplate reader (Spectra Max M5, Molecular Devices).

### The highly efficient redox reaction between KMnO_4_ and TMB

TMB solution was prepared by dissolving in ethanol. To verify the redox reaction between KMnO_4_ and TMB, the absorption spectra of 20 μM KMnO_4_, 200 μM TMB and their mixture between 350 nm and 750 nm were measured. To investigate the influence of pH on the redox reaction, the absorption spectra of KMnO_4_-TMB system prepared in 10 mM PBS buffer with different pH values (2, 2.5, 3, 3.5, 4, 4.5, 5, 5.5, 6, 6.5 and 7) were measured. To obtain the optimal concentration of TMB, the absorption spectra of KMnO_4_ (20 μM) after reaction with different concentrations of TMB (0, 25, 50, 100, 200, 250 and 500 μM) were measured. We also measured the absorption spectra of the corresponding solution after addition of sulfuric acid to further oxidize the oxidized TMB. Finally, to investigate the stability of the oxidized product, we also measured the absorption spectra of 20 μM KMnO_4_ solution after addition of 200 μM TMB for different times.

### The KMnO_4_-TMB system for H_2_O_2_ detection

To verify the reaction between KMnO_4_ and H_2_O_2_, the absorption spectra of 0.2 mM KMnO_4_ after reaction with different concentrations of H_2_O_2_ (0, 0.1, 1 and 10 mM) were measured. For the detection of H_2_O_2_, 8 μL of 0.5 mM KMnO_4_ was firstly reacted with different concentrations (0, 5, 10, 20, 40, 80, 100 and 200 μM) of H_2_O_2_ in 10 mM PBS buffer (pH 5.5) for 5 min. Then, 8 μL of 5 mM TMB was added into above solutions. The absorption spectra of these solutions between 350 nm and 750 nm were measured by microplate reader. The calibration curve for H_2_O_2_ sensing was made by using the absorbance intensity at 652 nm as the ordinate and the concentration of H_2_O_2_ as the abscissa.

### The KMnO_4_-TMB system for glucose detection

The KMnO_4_-TMB system also can be used for glucose detection by monitoring the generation of H_2_O_2_. For glucose detection, 4 μL of 0.1 mg mL^−1^ GOx was mixed with 50 μL of different concentrations of glucose in 0.5 mM PBS (pH 7.0). After incubated at 37 °C for 30 min, 130 μL of 10 mM PBS buffer (pH 5.5), 8 μL of 0.5 mM KMnO_4_ and 8 μL of 5 mM TMB were added into above solution. Then, the absorption spectra of these solutions between 350 nm and 750 nm were measured by microplate reader. The calibration curve for glucose sensing was made by using the absorbance intensity at 652 nm as the ordinate and the concentration of glucose as the abscissa. To investigate the specificity of this method, 1 mM other sugars such as sucrose, β-lactose, α-lactose and galactose, were used instead of 0.1 mM glucose under the same condition for glucose detection.

### Analysis of glucose in serum sample

The serum samples were obtained from Mengchao Hepatobiliary Hospital of Fujian Medical University, which was diluted by 0.5 mM PBS buffer (pH 7.0). The study was approved by the Medical Ethics Committee of Mengchao Hepatobiliary Hospital of Fujian Medical University, and the written consent was received from all participants in this study. The detection of glucose in serum sample was realized as follows: 4 μL of 0.1 mg mL^−1^ GOx was mixed with 50 μL of 100 times diluted serum and different concentrations of glucose in 0.5 mM PBS (pH 7.0). After incubated at 37 °C for 30 min, 122 μL of 10 mM PBS buffer (pH 5.5), 16 μL of 0.5 mM KMnO_4_ and 8 μL of 5 mM TMB were added into above solution. Then, the absorption spectra of these solutions between 350 nm and 750 nm were measured by microplate reader. The calibration curve for glucose sensing was made by using the absorbance intensity at 652 nm as the ordinate and the concentration of the added glucose as the abscissa. The added concentrations of glucose varied from 20 μM to 800 μM. The concentration of glucose in serum sample was estimated by the standard addition method.

## Results and discussion

### The highly efficient redox reaction between KMnO_4_ and TMB

**Fig. 1:**
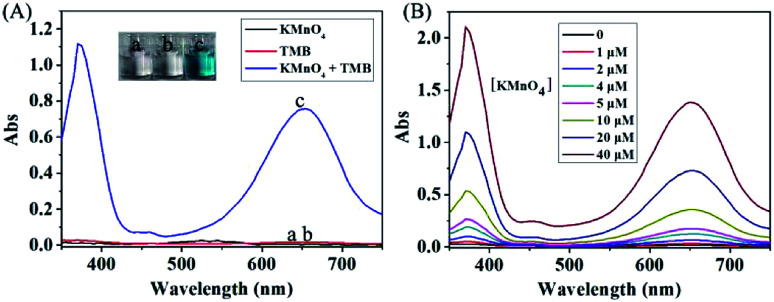
(A) The absorption spectra of different solutions: (a) 20 μM KMnO_4_; (b) 200 μM TMB; (c) 20 μM KMnO_4_ + 200 μM TMB. (B) The absorption spectra of different concentrations of KMnO_4_ after reaction with 200 μM TMB.

To verify the redox reaction between KMnO_4_ and TMB, the absorption spectra of KMnO_4_, TMB and their mixture were measured. As shown in Fig. 1A, KMnO_4_ and TMB themselves exhibited extremely low absorbance signal (curve a and b). However, upon the addition of TMB, the KMnO_4_ solution had significant absorbance enhancement at 652 nm (curve c), which is consistent with the characteristic absorption peak of oxidized TMB. This result indicated that KMnO_4_ can directly induce the oxidation of TMB to generate the oxidized product with significant color change. This reaction can be also confirmed by the photograph of corresponding solution shown in Fig. 1A.

In order to obtain the best reaction efficiency, we optimized the reaction condition. To investigate the influence of pH on the redox reaction, the absorption spectra of KMnO_4_-TMB system prepared in 10 mM PBS buffer with different pH values were measured. As shown in Fig. S1 in the ESI,† the reaction efficiency was obviously affected by the pH value of the buffer. In more acidic conditions, the absorption peak of the KMnO_4_-TMB system shifted to 450 nm, which may be due to the further oxidation of oxidized TMB. It is reported that the oxidized TMB can be further oxidized to a yellow diimine after addition of sulfuric acid, with the maximum absorption peak at 450 nm. This process can be also confirmed by the results shown in Fig. S1B in the ESI.† The best pH value of PBS buffer is 5.5 due to its best absorbance signal.

To optimize the concentration of TMB, the absorption spectra of KMnO_4_ after reaction with different concentrations of TMB were measured in the optimized buffer. As shown in Fig. S2 in the ESI,† TMB at low concentration could be partly oxidized to yellow oxide. When the concentration of TMB up to 200 μM, TMB could be mainly oxidized to blue oxide. While further increase the concentration of TMB, the absorbance signal might slightly decrease. After comparison, the optimal concentration of TMB is 200 μM due to its best absorbance signal. We also investigate the stability of the oxidized product by measuring the absorption spectra of KMnO_4_ solution after addition of 200 μM TMB for different times. As shown in Fig. S3 in the ESI,† there is no significant difference in the absorbance signals at different times. This data indicated that the redox reaction between KMnO_4_ and TMB is very fast, and the experimental result can be stable for a relatively long period, which have great advantages in practical applications.

In the optimized condition, we measured the absorption spectra of different concentrations of KMnO_4_ solution after reaction with 200 μM TMB. As shown in Fig. 1B, the absorbance signal increased by increasing the concentration of KMnO_4_. This redox reaction is highly efficient due to that 1 μM of KMnO_4_ was enough to cause the detectable change in absorbance signal.

### The KMnO_4_-TMB system for H_2_O_2_ detection

**Fig. 2:**
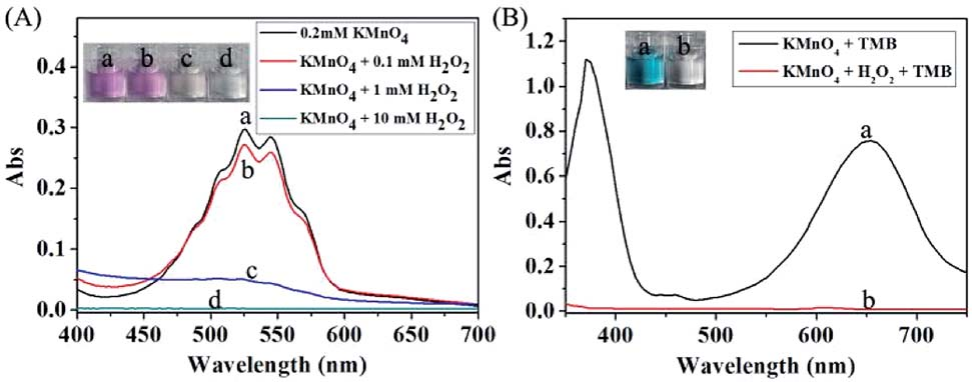
(A) The absorption spectra of 0.2 mM KMnO_4_ after reaction with different concentrations of H_2_O_2_. (B) The absorption spectra of different solutions: (a) 20 μM KMnO_4_ + 200 μM TMB; (b) 20 μM KMnO_4_ + 0.5 mM H_2_O_2_ + 200 μM TMB.

It is well known that H_2_O_2_ is able to reaction with KMnO_4_ in acidic condition, and thus causing the consumption of KMnO_4_. In this way, the KMnO_4_-TMB system can be used for the detection of H_2_O_2_. We first used the absorbance data to verify the reaction between KMnO_4_ and H_2_O_2_. KMnO_4_ has two large absorption peaks at 525 nm and 545 nm, and its solution is purple. After reaction with H_2_O_2_, the absorbance of the solution obviously reduced, accompanied by the color fading to colorless (Fig. 2A). This phenomenon can confirm the reaction between KMnO_4_ and H_2_O_2_. Based on this reaction, the KMnO_4_-TMB system could be used for the detection of H_2_O_2_.

**Fig. 3:**
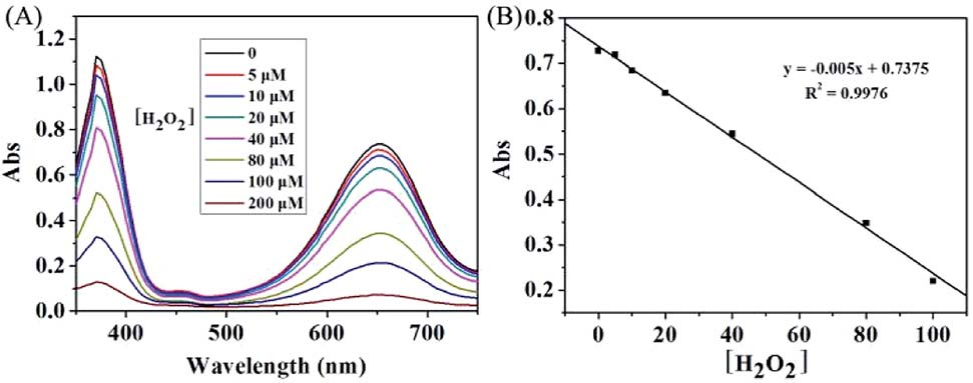
(A) The absorption spectra of the KMnO_4_-TMB system for analyzing different concentrations of H_2_O_2_. (B) Calibration curve of H_2_O_2_ in the range from 5 to 100 μM. The data shown here represent the average of three independent experiments.

As shown in Fig. 2B, the KMnO_4_-TMB system exhibited high absorbance at 652 nm. When H_2_O_2_ was introduced, the absorbance intensity of the solution significantly reduced, accompanied by the color fading from blue to colorless. We also measured the absorption spectra of the KMnO_4_-TMB system for analyzing different concentrations of H_2_O_2_. As shown in Fig. 3, the absorbance intensity of the system gradually decreased by increasing the concentration of added H_2_O_2_. Meanwhile, the absorbance intensity at 652 nm was linear to the concentration of H_2_O_2_ in the range from 5 to 100 μM, with a calibration function of *y* = −0.005*x* + 0.7375 (*R*^2^ = 0.9976). This redox reaction based method permits detection of 5 μM H_2_O_2_.

### The KMnO_4_-TMB system for glucose detection

**Fig. 4:**
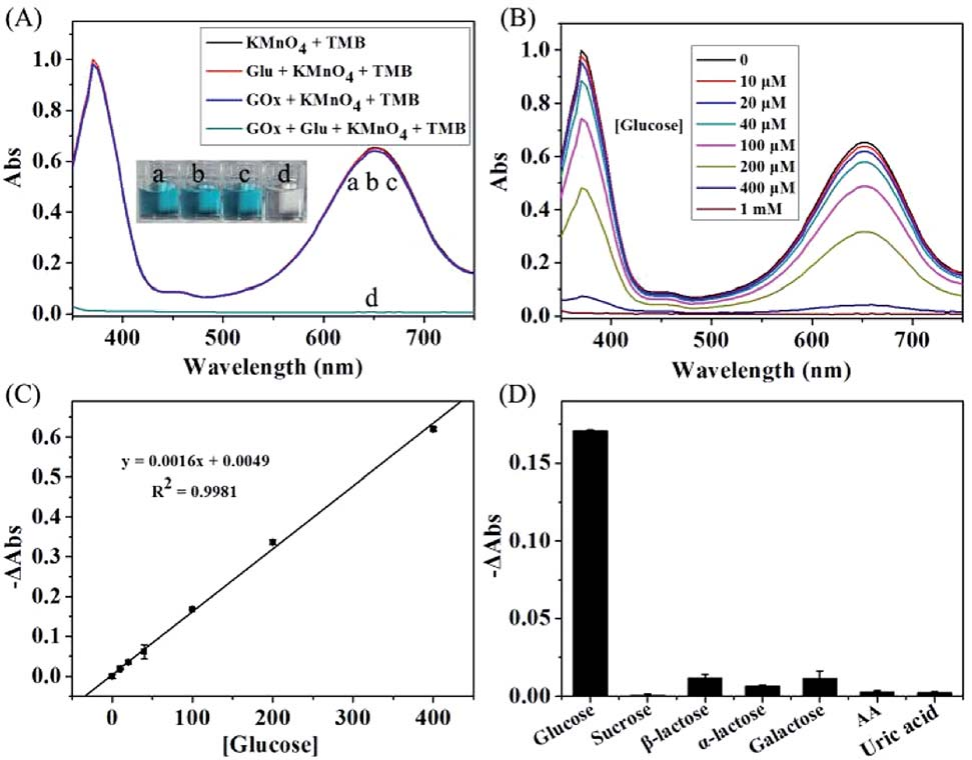
(A) The absorption spectra of KMnO_4_-TMB system upon addition of the below solution: (a) control; (b) glucose (1 mM); (c) GOx (2 μg mL^−1^); (d) glucose reacted with GOx for 30 min. (B) The absorption spectra of the KMnO_4_-TMB system for analyzing different concentrations of glucose. (C) Calibration curve of glucose in the range from 10 to 400 μM. (D) Absorbance changes (−ΔAbs) of the system for analyzing glucose (100 μM), other sugars (sucrose, β-lactose, α-lactose and galactose, each at 1 mM) and other interferences (ascorbic acid and uric acid, each at 100 μM). The data shown here represent the average of three independent experiments.

The KMnO_4_-TMB system also can be used for the indirectly detection of other biomolecules by monitoring the generation or consumption of H_2_O_2_. It is well known that GOx can catalyze the oxidation of glucose in the presence of oxygen, resulting in the formation of H_2_O_2_. Thus, the detection of glucose could be realized by using the KMnO_4_-TMB system. We first use the absorbance data to investigate the viability of this method. As shown in Fig. 4A, the KMnO_4_-TMB system exhibited high absorbance at 652 nm. When only addition of glucose or GOx, the absorbance signal of the system was little influenced. If glucose was first reacted with GOx and then introduced to the KMnO_4_-TMB system, the absorbance of the solution significantly reduced, accompanied by the color fading from blue to colorless. This phenomenon confirmed that the KMnO_4_-TMB system can be used for the detection of glucose.

We then measured the absorption spectra of the KMnO_4_-TMB system for analyzing different concentrations of glucose. As shown in Fig. 4B, the KMnO_4_-TMB system exhibited high absorbance at 652 nm when only addition of GOx. When glucose was introduced, the absorbance intensity of the system slowly decreased by increasing the concentration of added glucose. Meanwhile, the reduced absorbance intensity at 652 nm was linear to the concentration of glucose in the range from 10 to 400 μM, with a calibration function of *y* = 0.0016*x* + 0.0049 (*R*^2^ = 0.9981). The detection limit of this method was calculated to be 4.55 μM, which gave a net signal equal to three times the standard deviation of the background. As shown in Table S1 in the ESI,† this sensitivity is comparable to many other nanomaterials based methods, but avoiding the preparation and storage of the nanomaterials.^6,9,26–34^

To investigate the specificity of this method, 1 mM other sugars (sucrose, β-lactose, α-lactose and galactose) were used instead of 0.1 mM glucose under the same condition for glucose detection. As shown in Fig. 4C, the system showed obvious absorbance decrease when analyzing 0.1 mM of glucose. While, the absorbance signals of the system were scarcely influenced by other sugars even at ten times of the concentration of glucose. The other interferences, such as ascorbic acid and uric acid, have also been investigated. The absorbance signals of the system were scarcely changed, when GOx was added to initiate the reaction. Thus, the proposed KMnO_4_-TMB system can show high selectivity for glucose detection.

### Analysis of glucose in real sample

**Fig. 5:**
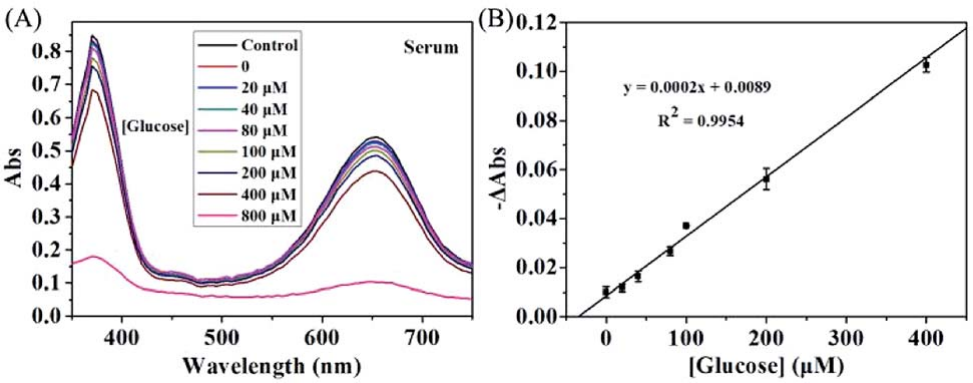
The absorption spectra of the KMnO_4_-TMB system for analyzing diluted serum with addition of different concentrations of glucose (0, 20, 40, 80, 100, 200, 400 and 800 μM, respectively). (B) Calibration curve of glucose sensing in diluted serum using the reduced absorbance intensity (−ΔAbs) as the ordinate and the concentrations of added glucose as the abscissa. The data shown here represent the average of three independent experiments.

The concentration of glucose in blood is closely associated with hypoglycemia or diabetes, which can be also used for monitoring many other diseases. Thus, accurate measurement of glucose is of great importance. To investigate the feasibility of KMnO_4_-TMB system for practical application, we further use the proposed method for analyzing glucose in serum sample, which obtained from Mengchao Hepatobiliary Hospital of Fujian Medical University. Fig. 5A showed the absorption spectra of the KMnO_4_-TMB system for analyzing diluted serum with addition of GOx and different concentrations of glucose. The absorbance signal of the system slowly decreased by increasing the concentration of spiked glucose. Meanwhile, the reduced absorbance intensity at 652 nm was linear to the concentration of spiked glucose, with a calibration function of *y* = 0.0002*x* + 0.0089 (*R*^2^ = 0.9954). We then used a standard addition method to estimate the concentration of glucose in serum. After calculation, the concentration of glucose in diluted serum was 44.5 μM. Thus, the actual concentration of glucose in serum sample was 4.45 mM, which agreed with the fact that the normal level of glucose in blood is between 3.9 and 6.1 mM.^35^ It is worth mentioning that the GOx does not affect the redox reaction between KMnO_4_ and TMB, when the concentration of GOx was lower than 2 μg mL^−1^. Meanwhile, 10 ng mL^−1^ of GOx was enough to cause the detectable absorbance signal (Fig. S4 in the ESI†). Therefore, the KMnO_4_-TMB system can be also expected to be used in immunoassays by combining antibody and GOx, and thus further expand its applications.

## Conclusions

In this study, we have designed a novel KMnO_4_-TMB system for H_2_O_2_ based colorimetric assay based on the KMnO_4_ induced oxidation of TMB, that could generate oxidized blue product. The designed system is very fast, stable and highly efficient due to that 1 μM of KMnO_4_ is enough to induce the detectable change in absorbance signal. The current system can be used for the detection of H_2_O_2_ by the consumption of KMnO_4_, and also can be used for the detection of glucose even in serum sample by monitoring the generation of H_2_O_2_, which permits detection of as low as 10 μM glucose that is comparable to or lower than most peroxidase mimetic based methods, but avoiding the preparation and storage of the nanomaterials. Taking together, this highly efficient redox reaction between KMnO_4_ and TMB might be further expected to food safety, environmental analysis, clinical diagnosis and other fields.

## Conflicts of interest

There are no conflicts to declare.

## Supplementary Material

RA-009-C8RA07758D-s001

## References

[cit1] Wei H., Wang E. (2013). Chem. Soc. Rev..

[cit2] Lin Y., Ren J., Qu X. (2014). Acc. Chem. Res..

[cit3] Wang Q., Wei H., Zhang Z., Wang E., Dong S. (2018). TrAC, Trends Anal. Chem..

[cit4] Gao L., Zhuang J., Nie L., Zhang J., Zhang Y., Gu N., Wang T., Feng J., Yang D., Perrett S., Yan X. (2007). Nat. Nanotechnol..

[cit5] Sun X., Guo S., Chung C. S., Zhu W., Su S. (2013). Adv. Mater..

[cit6] Song Y., Qu K., Zhao C., Ren J., Qu X. (2010). Adv. Mater..

[cit7] Wang Q., Lei J., Deng S., Zhang L., Ju H. (2013). Chem. Commun..

[cit8] Zheng A. X., Cong Z. X., Wang J. R., Li J., Yang H. H., Chen G. N. (2013). Biosens. Bioelectron..

[cit9] Chen H., Qiu Q., Sharif S., Ying S., Wang Y., Ying Y. (2018). ACS Appl. Mater. Interfaces.

[cit10] Cheng H., Liu Y., Hu Y., Ding Y., Lin S., Cao W., Wang Q., Wu J., Muhammad F., Zhao X., Zhao D., Li Z., Xing H., Wei H. (2017). Anal. Chem..

[cit11] Tian Z., Li J., Zhang Z., Gao W., Zhou X., Qu Y. (2015). Biomaterials.

[cit12] Han L., Li C., Zhang T., Lang Q., Liu A. (2015). ACS Appl. Mater. Interfaces.

[cit13] Lu C., Liu X., Li Y., Yu F., Tang L., Hu Y., Ying Y. (2015). ACS Appl. Mater. Interfaces.

[cit14] Jiang X., Sun C., Guo Y., Nie G., Xu L. (2015). Biosens. Bioelectron..

[cit15] Zheng A., Zhang X., Gao J., Liu X., Liu J. (2016). Anal. Chim. Acta.

[cit16] Wang Y. W., Tang S., Yang H. H., Song H. (2016). Talanta.

[cit17] Wang W., Wang L., An F., Xu H., Yin Z., Tang S., Yang H. H., Song H. (2017). Anal. Chim. Acta.

[cit18] Ganganboinaa A. B., Doonga R. (2018). Sens. Actuators, B.

[cit19] Pal J., Pal T. (2016). RSC Adv..

[cit20] Lai W., Zeng Q., Tang J., Zhang M., Tang D. (2018). Microchim. Acta.

[cit21] Ji D., Du Y., Meng H., Zhang L., Huang Z., Hu Y., Li J., Yu F., Li Z. (2018). Sens. Actuators, B.

[cit22] Ding Y., Zhao J., Li B., Zhao X., Wang C., Guo M., Lin Y. (2018). Microchim. Acta.

[cit23] Liu J. W., Luo Y., Wang Y. M., Duan L. Y., Jiang J. H., Yu R. Q. (2016). ACS Appl. Mater. Interfaces.

[cit24] Ding L., Gong Z., Yan M., Yu J., Song X. (2017). Microchim. Acta.

[cit25] Nichols S. P., Koh A., Storm W. L., Shin J. H., Schoenfisch M. H. (2013). Chem. Rev..

[cit26] Wei H., Wang E. (2008). Anal. Chem..

[cit27] Hu L., Yuan Y., Zhang L., Zhao J., Majeed S., Xu G. (2013). Anal. Chim. Acta.

[cit28] Liu X., Zhang S., Tan P., Zhou J., Huang Y., Nie Z., Yao S. (2013). Chem. Commun..

[cit29] Jv Y., Li B., Cao R. (2010). Chem. Commun..

[cit30] Liu J., Hu X., Hou S., Wen T., Liu W., Zhu X., Yin J. J., Wu X. (2012). Sens. Actuators, B.

[cit31] Lin T., Zhong L., Guo L., Fu F., Chen G. (2014). Nanoscale.

[cit32] Chen T. M., Wu X. J., Wang J. X., Yang G. W. (2017). Nanoscale.

[cit33] Li M., Liu L., Shi Y., Yang Y., Zheng H., Long Y. (2017). New J. Chem..

[cit34] Wang Y. M., Liu J. W., Jiang J. H., Zhong W. (2017). Anal. Bioanal. Chem..

[cit35] Ma J. L., Yin B. C., Wu X., Ye B. C. (2016). Anal. Chem..

