# Supplementary material for: Highly efficient redox reaction between potassium permanganate and 3,3′,5,5′-tetramethylbenzidine for application in hydrogen peroxide based colorimetric assays
Source: RSC Adv. 2019 Jan 14;9(4):1889–94. doi: 10.1039/c8ra07758d (PMC9059746; doi:10.1039/c8ra07758d)
Supplement: RA-009-C8RA07758D-s001 [file RA-009-C8RA07758D-s001.pdf]

Electronic Supplementary Material (ESI) for RSC Advances.
This journal is © The Royal Society of Chemistry 2019

Electronic Supplementary Information

Highly efficient redox reaction between potassium permanganate and 3, 3’, 5, 5’-tetramethylbenzidine for application in hydrogen peroxide based colorimetric assay

Ying Sun,[abc] Hui Liu,[e] Xionghong Tan,[abc] Zheng Li,[d] Yanlin Du,[abc] Aixian Zheng,*[bc] Xiaolong Liu*[bc] and Niancai Peng*[d]

[a.] College of Life Science, Fujian Agriculture and Forestry University, Fuzhou, Fujian 350002, P. R. China.

[b.] Fujian Institute of Research on the Structure of Matter, Chinese Academy of Sciences, Fuzhou, Fujian 350002, P. R. China.

[c.] The United Innovation of Mengchao Hepatobiliary Technology Key Laboratory of Fujian Province, Mengchao Hepatobiliary Hospital of Fujian Medical University, Fuzhou 350025, P. R. China.

[d.] State Key Laboratory for Manufacturing System Engineering, School of Mechanical Engineering, Xi'an Jiaotong University, Xi'an 710049, P. R. China.

[e.] Fifth People’s Hospital, Ganzhou City, Jiangxi Province.

*Corresponding authors.

Tel: +86-591-83705927; E-mail addresses:

zax040500273@126.com; xiaoloong.liu@gmail.com; pnc@mail.xjtu.edu.cn.

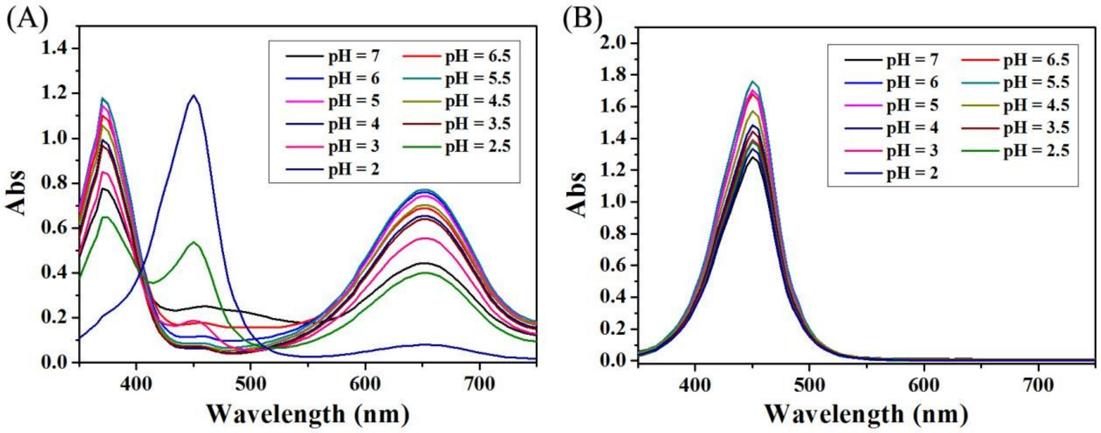

Fig. S1 (A) The absorption spectra of $KMnO_4$-TMB system performed in 10 mM PBS buffer with different pH values (2, 2.5, 3, 3.5, 4, 4.5, 5, 5.5, 6, 6.5 and 7); (B) the absorption spectra of the corresponding solution after addition of sulfuric acid.

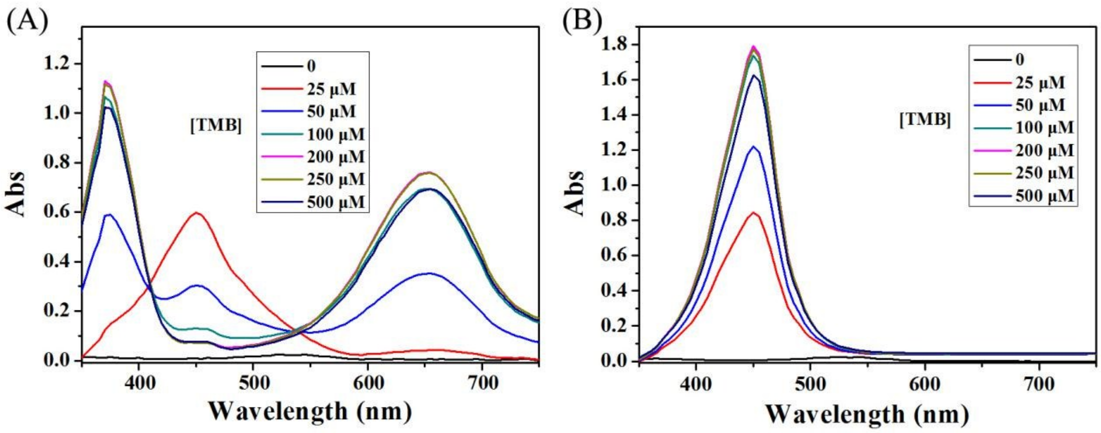

Fig. S2 (A) The absorption spectra of $KMnO_4$ (20 μM) after reaction with different concentrations of TMB (0, 25, 50, 100, 200, 250 and 500 μM); (B) the absorption spectra of the corresponding solution after addition of sulfuric acid.

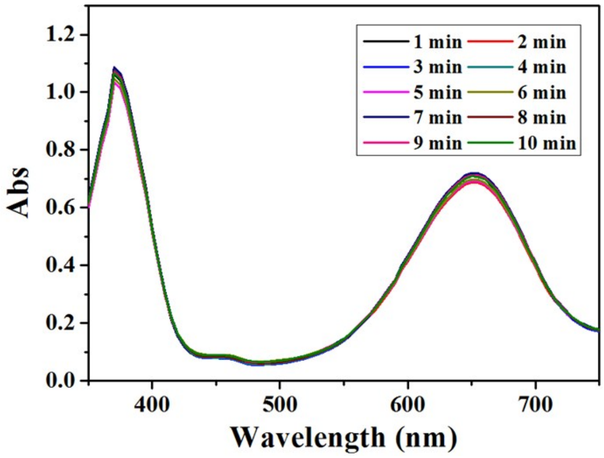

Fig. S3 The absorption spectra of 20 μM $KMnO_4$ solution after addition of 200 μM TMB for different times.

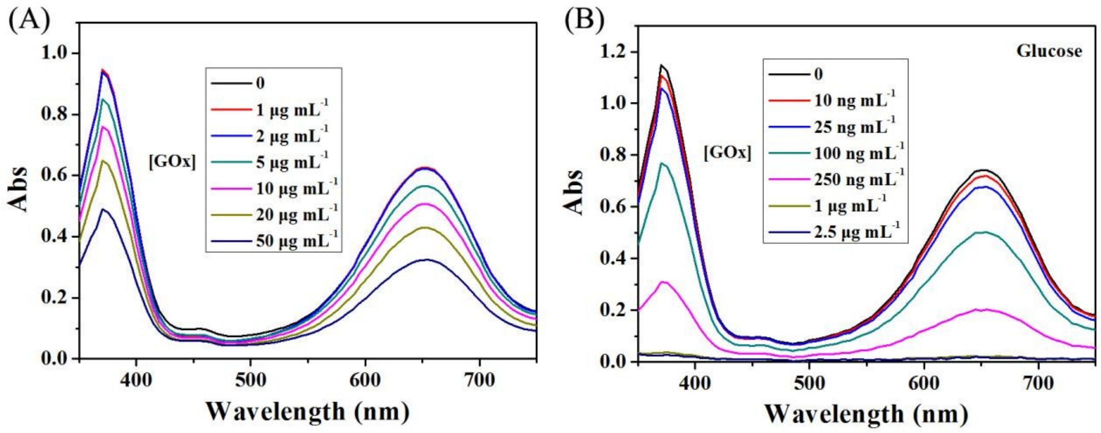

Fig. S4 (A) The absorption spectra of $KMnO_4$-TMB system upon addition of different concentrations of GOx (varied from 1 μg mL^{-1} to 50 μg mL^{-1}); (B) The absorption spectra of $KMnO_4$-TMB system for glucose detection by using different concentrations of GOx (varied from 10 ng mL^{-1} to 2.5 μg mL^{-1}).

Table S1 Comparison of the present method with other methods for the detection of glucose.

Materials	Linear range (μM)	LOD (μM)	Reference
Fe_3O_4 MNPs	50-1000	30	1
Graphene Oxide	1-20	1	2
Cu NCs	100-2000	100	3
Gold nanorods	100-1000	100	4
AuNPs	18-1100	4	5
Au@Pt core-shell nanorods	45-400	45	6
MoS_2 Nanosheets	5-150	1.2	7
WSe_2 Nanosheets	10-60	10	8
g-C_3N_4 nanosheets	-	0.4	9
Dichlorofluorescein	80-1200	30	10
PdNPs/Cu-TCPP(Fe)	2-200	0.994	11
CoOOH nanoflakes	5.3-100	5.3	12
$KMnO_4$ - TMB	10-400	4.55	This work

References

1. H. Wei, E. Wang, *Anal. Chem.*, 2008, **80**, 2250-2254.

2. Y. Song, K. Qu, C. Zhao, J. Ren, X. Qu, *Adv. Mater.*, 2010, **22**, 2206-2210.

3. L. Hu, Y. Yuan, L. Zhang, J. Zhao, S. Majeed, G. Xu, *Anal. Chim. Acta.*, 2013, **762**, 83-86.

4. X. Liu, S. Zhang, P. Tan, J. Zhou, Y. Huang, Z. Nie, S. Yao, *Chem. Commun.*, 2013, **49**, 1856-1858.

5. Y. Jv, B. Li, R. Cao, *Chem. Commun.*, 2010, **46**, 8017-8019.

6. J. Liu, X. Hu, S. Hou, T. Wen, W. Liu, X. Zhu, J. J. Yin, X. Wu, *Sensors and Actuators B*, 2012, **166**, 708-714.

7. T. Lin, L. Zhong, L. Guo, F. Fu, G. Chen, *Nanoscale*, 2014, **6**, 11856-11862.

8. T. M. Chen, X. J. Wu, J. X. Wang, G. W. Yang, *Nanoscale*, 2017, **9**, 11806-11813.

9. J. W. Liu, Y. Luo, Y. M. Wang, L. Y. Duan, J. H. Jiang, R. Q. Yu, *ACS Appl. Mater. Interfaces*, 2016, **8**, 33439-33445.

10. M. Li, L. Liu, Y. Shi, Y. Yang, H. Zheng, Y. Long, *New J. Chem.*, 2017,**41**, 7578-7582

11. H. Chen, Q. Qiu, S. Sharif, S. Ying, Y. Wang, Y. Ying, *ACS Appl. Mater. Interfaces*, 2018, **10**, 24108-24115.

12. Y. M. Wang, J. W. Liu, J. H. Jiang, W. Zhong, *Anal Bioanal Chem*, 2017, **409**, 225-4232.
